# Rapid On-Site Detection of *Colletotrichum gloeosporioides* Using EASY DNA Extraction (EZ-D) Method Combined with RPA-CRISPR/Cas12a

**DOI:** 10.3390/plants15101565

**Published:** 2026-05-20

**Authors:** Chun Yang, Size Dai, Bolin Wang, Jiahui Zang, Yuzhe Kong, Chao Chen, Haiwen Wang, Tingting Dai

**Affiliations:** 1 Co-Innovation Center for the Sustainable Forestry in Southern China, Nanjing Forestry University, Nanjing 210037, China; yangchun0724@163.com (C.Y.); zjh20011009@163.com (J.Z.); 18014817696@163.com (Y.K.); chaochen0520@163.com (C.C.); 13865744264@163.com (H.W.); 2 Department of Customs Inspection and Quarantine, Shanghai Customs University, Shanghai 200120, China; 18260036961@139.com; 3 College of Information Science and Technology & College of Artificial Intelligence, Nanjing Forestry University, Nanjing 210037, China; 1955665910@njfu.edu.cn

**Keywords:** CRISPR/Cas12a, *Colletotrichum gloeosporioides*, RPA, EZ-D rapid extraction, POCT

## Abstract

Anthracnose, caused by *Colletotrichum gloeosporioides*, is a globally distributed phytopathogenic disease with a broad host range, posing a serious threat to the healthy growth of forest trees, including *Cunninghamia lanceolata*. To enable rapid and accurate on-site detection of this pathogen, this study developed a comprehensive field-deployable detection method. The approach integrates the EZ-D method (EASY DNA extraction) for rapid nucleic acid extraction with recombinase polymerase amplification (RPA) and the CRISPR/Cas12a system. A specific target gene, designated *Cglo6922*, was identified for the detection of *C. gloeosporioides*. The entire detection process can be completed within approximately 25 min, comprising a 10-min isothermal RPA at 39 °C followed by a 15-min Cas12a cleavage reaction. Specificity evaluation showed that the method successfully detected two *C. gloeosporioides* isolates derived from different hosts, while no cross-reactivity was observed against a panel of 32 other isolates, including ten *Colletotrichum* species, eight *Phytophthora* species, six *Pythium* species, seven *Fusarium* species, and one *Botryosphaeria dothidea* isolate, demonstrating robust species-level specificity. Sensitivity testing revealed that the method achieved a limit of detection (LOD) of 10 pg/μL of genomic DNA for *C. gloeosporioides*. Furthermore, by incorporating the EZ-D rapid extraction method (requiring only one minute for DNA extraction at a cost of approximately $0.03 USD per sample), target nucleic acid was successfully extracted from artificially inoculated *Cunninghamia lanceolata* branch samples and proved compatible with the RPA-CRISPR/Cas12a detection system. In conclusion, this study establishes a novel field-deployable detection method for *C. gloeosporioides* that is rapid, cost-effective, highly specific, and highly sensitive, providing a powerful tool for point-of-care testing (POCT) of this disease.

## 1. Introduction

*Cunninghamia lanceolata* (Lamb.) Hook. (Chinese Fir) is an important fast-growing timber species native to China. It possesses one of the largest afforestation areas and timber volumes among both domestic and worldwide planted coniferous forests, and plays a significant role in forest carbon sequestration, increasing forest farmer income, and rural revitalization in China [1,2]. Anthracnose is one of the major fungal diseases of *Cunninghamia lanceolata*. It is widely distributed and causes severe damage in *Cunninghamia lanceolata* cultivation areas, posing a serious threat to the healthy development of the *Cunninghamia lanceolata* industry. The primary causal agent of this disease is *Colletotrichum gloeosporioides*, a fungus belonging to the phylum Ascomycota, class Sordariomycetes, family Glomerellaceae, genus *Colletotrichum* [2,3,4]. This pathogen causes severe damage worldwide, infecting hundreds of host plant species across dozens of families, including forest trees, fruit trees, ornamentals, and field crops, leading to serious anthracnose diseases, substantial economic losses, and ecological damage [5,6,7]. As a broad-spectrum phytopathogenic fungus, *C. gloeosporioides* has a host range encompassing both woody and herbaceous economic crops, infecting over 470 genera of monocotyledonous or dicotyledonous plants [8,9]. For example, it has been reported that *C. gloeosporioides* isolates from citrus could have a latent period of several weeks, and their morphological and physiological characteristics were variable compared to those of isolates from mango [10]. *C. gloeosporioides* isolates from rubber trees are genetically very close to apple anthracnose strains (over 99% rDNA-ITS similarity) [11], exhibit a long latent period, and display high genotypic diversity [12,13]. Given this prolonged latent period—up to several weeks in citrus—which delays symptom expression [14], a rapid on-site detection method for *C. gloeosporioides* is urgently needed. Traditional laboratory detection methods are time-consuming (taking 2–3 days) and often result in missed optimal windows for disease control. Rapid, field-based identification of the pathogen would enable early warning and precise fungicide application, thereby reducing economic losses and unnecessary pesticide use [15]. Moreover, *C. gloeosporioides* is morphologically similar to closely related species and shares high genetic similarity with them (e.g., over 99% rDNA-ITS identity with *Colletotrichum acutatum*), making it difficult to distinguish using conventional methods. Therefore, developing a detection method that combines high specificity with field adaptability is particularly urgent. These features complicate the differentiation of *C. gloeosporioides* from closely related *Colletotrichum* species using conventional methods. Therefore, a rapid, specific, and field-deployable detection method capable of distinguishing this pathogen from its close relatives is urgently needed.

Currently, traditional morphological identification methods are time-consuming [16], have low sensitivity, require highly skilled personnel [17], and often yield inaccurate results [18]. For molecular detection of *C. gloeosporioides*, various specific detection techniques have been developed, including conventional PCR and real-time quantitative PCR (qPCR). In the citrus host system, a multiplex PCR assay using species-specific primers targeting the GAPDH gene was shown to successfully distinguish *C. abscissum* from *C. gloeosporioides* sensu stricto, offering a rapid and economical tool for diagnosing post-bloom fruit drop (PFD) of citrus [19]. For strawberry (*Fragaria*) hosts, a real-time qPCR system targeting the ITS region was developed, allowing specific detection of *C. gloeosporioides* and *C. acutatum*. Compared to conventional PCR, this system achieved a 100-fold increase in sensitivity for *C. acutatum* (detection limit equivalent to DNA from 7.2 conidia) and a 10-fold increase for *C. gloeosporioides* [20]. For the apple (Malus) host system, species-specific TaqMan qPCR assays were developed targeting nine Colletotrichum species that cause bitter rot, including *C. gloeosporioides* sensu stricto. The primers and probes for *C. gloeosporioides* s.s. were designed based on the GAPDH gene, achieving a detection sensitivity of 0.5 pg of DNA, and four primer-probe sets were subsequently optimized for duplex detection [21]. For oil-tea camellia (*Camellia oleifera*) anthracnose, a qPCR method targeting the specific gene v012077 was developed to detect *C. fructicola* (a member of the *C. gloeosporioides* species complex), achieving a sensitivity of a single conidium (equivalent to 0.063 pg of DNA) [22]. For citrus leaves, a multiplex real-time qPCR system was developed to simultaneously detect and quantify *C. abscissum* and *C. gloeosporioides*, offering a high-throughput tool for field pathogen monitoring [23].

However, these techniques have limitations, such as long reaction times (approximately 2–3 h for qPCR, including nucleic acid extraction, PCR amplification, and data analysis), and the need for specialized equipment, which restrict their application in field disease diagnosis [24,25,26,27]. To address these issues, researchers have developed and applied isothermal amplification-based molecular detection techniques, such as loop-mediated isothermal amplification (LAMP) and recombinase polymerase amplification (RPA) [12,28]. These techniques offer the advantages of rapidity, high specificity, and high sensitivity because they amplify nucleic acids under constant temperature conditions without the need for thermal cycling equipment. Recombinase polymerase amplification (RPA), developed by Piepenburg et al. in 2006, offers a promising approach for the rapid amplification of target nucleic acids from minimally processed samples [29]. With its compact and portable instrumentation, simple operation, rapid amplification speed, and isothermal reaction conditions at 37–42 °C, RPA shows potential as a viable alternative to PCR. Since its development in 2006, RPA has gained significant traction in various detection applications, including bacteria, fungi, and viruses, particularly over the past decade [30].

To further enhance the specificity and visualization capability of detection systems, researchers have combined the CRISPR-Cas system with RPA technology. The CRISPR-Cas system, originally discovered as an adaptive immune system in prokaryotes, defends against invasive genetic elements by acquiring novel spacers and activating sequence-specific nuclease activity [31,32]. Cas12a orthologs specifically recognize a short PAM sequence located upstream of the target sequence, thereby activating their trans-cleavage capability [33,34,35]. Specifically, Cas12a and CRISPR RNA (crRNA) form a ribonucleoprotein (RNP) complex, recognize the protospacer adjacent motif (PAM) site with the “TTTN” motif, and bind to double-stranded DNA complementary to the crRNA, forming a ternary complex [36]. This specific recognition subsequently activates its trans-cleavage activity, enabling the non-specific cleavage of labeled single-stranded DNA reporter probes bearing a fluorophore-quencher pair. Once the probe is cleaved by the Cas protein, the quencher is removed, and the reporter fluorophore emits fluorescence, enabling visual detection [37].

Efficient and rapid nucleic acid extraction is fundamental to molecular biology and is a critical prerequisite for achieving on-site field detection. The traditional CTAB (cetyltrimethylammonium bromide) method, established by Doyle and Doyle, is one of the most classic and widely used methods for plant DNA extraction [38]. However, the CTAB method has significant limitations: the procedure is cumbersome and time-consuming, requiring approximately 2 h for a complete process involving over 20 steps; it requires hazardous chemicals such as liquid nitrogen, phenol, and chloroform, posing biosafety risks; the use of organic solvents generates hazardous waste requiring special treatment and disposal; high-throughput processing is difficult; and it requires considerable technical expertise [39]. To address these issues and meet the demand for simple, rapid, and safe nucleic acid extraction for field applications, various rapid extraction methods have been developed, including those based on silica membranes, magnetic beads, Chelex-100, microneedle patches, and filter paper combined with LAMP [40]. Each method has its own advantages and limitations. For example, silica membrane-based methods provide high-purity DNA but require multiple centrifugation steps and specialized kits, increasing cost and operation time [41]. Magnetic bead-based methods enable high-throughput processing and are easily automated, but they rely on expensive beads and magnetic separators. Chelex-100 extraction is simple and inexpensive, involving only a boiling step, yet the DNA yield is often low and contains PCR inhibitors that may affect downstream amplification [42]. The microneedle patch-based approach enables DNA extraction within one minute, but it has limitations including relatively high impurity levels, elevated production costs for large-scale manufacturing, the requirement for cleanroom facilities, and unverified generalizability [43]. The filter paper extraction method combined with LAMP allows detection within 20 min without specialized equipment; however, it faces challenges such as complex primer design, high sensitivity that increases the risk of product contamination (10^9^ copies produced within one hour), stringent anti-contamination requirements, and a high demand for field validation [44,45]. In contrast, the EZ-D (EASY DNA extraction) method, as a more balanced solution, was modified and named in a previous study. It is important to note that the “EZ-D method” used in this study is not a commercial “EZ DNA extraction kit” (such as those from Zymo Research), but rather a self-developed rapid nucleic acid extraction method based on filter paper and PVC sheets, with its name derived from the abbreviation of “EASY DNA extraction.” This method involves attaching cellulose filter paper to a polyvinyl chloride (PVC) sheet for sample processing [46]. It offers core advantages including rapid operation (30–60 s), low cost (approximately 0.2 RMB per sample), no requirement for organic reagents or specialized equipment, high extraction efficiency, and good stability [47]. More importantly, the DNA purity achieved using this method is sufficient to support various amplification methods, including conventional PCR, LAMP, and RPA, making it fully adaptable for field conditions. Additionally, a lateral flow device (LFD)-based method has been developed for on-site detection, which involves disrupting samples using a metal bead and then transferring the lysate to a nitrocellulose membrane [48]. The DNA captured on the membrane exhibits good stability at room temperature, facilitating field operations.

**Figure 1:**
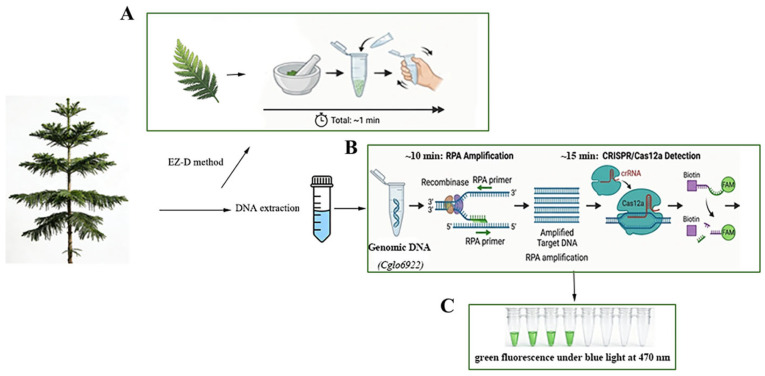
Schematic diagram of the EZ-D-RPA-CRISPR/Cas12a assay for the detection of *C. gloeosporioides*. (**A**) Rapid DNA extraction using the EZ-D method. Approximately 5–10 mg of leaf tissue is ground with extraction buffer E for 10 s. The EZ-D paper strip is immersed for 10 s, washed for 5 s, and eluted for 5 s. No centrifuge or specialized equipment is required. (**B**) RPA (39 °C, 10 min) followed by CRISPR/Cas12a detection (39 °C, 15 min). The Cas12a/crRNA complex specifically recognizes the target amplicon, activating non-specific trans-cleavage of the FAM-BHQ1 labeled ssDNA reporter. (**C**) Positive results are visualized as green fluorescence under a blue LED transilluminator (470 nm).

In summary, although the RPA-CRISPR/Cas12a isothermal amplification technology and the EZ-D rapid nucleic acid extraction method have demonstrated excellent performance in the detection of various pathogens, As of the time of submission, no study has reported the integration of the RPA-CRISPR/Cas12a system with the EZ-D method for the rapid field detection of *C. gloeosporioides*. Although some LAMP-based methods exist, they have limitations in sensitivity, speed, and field deployability. This is precisely where the novelty of our study lies. Therefore, this study combined the EZ-D method for rapid on-site sample nucleic acid extraction and established an efficient detection system for *C. gloeosporioides* based on RPA-CRISPR/Cas12a (Figure 1). This detection method is simple to operate, short in duration, highly specific and sensitive, and applicable for point-of-care testing in the field, providing strong technical support for the rapid field diagnosis of *C. gloeosporioides* and early disease prevention and control.

## 2. Results

### 2.1. Selection of Extraction Solution for Rapid DNA Extraction of C. gloeosporioides Using the EZ-D Method and Cost Comparison

**Figure 2:**
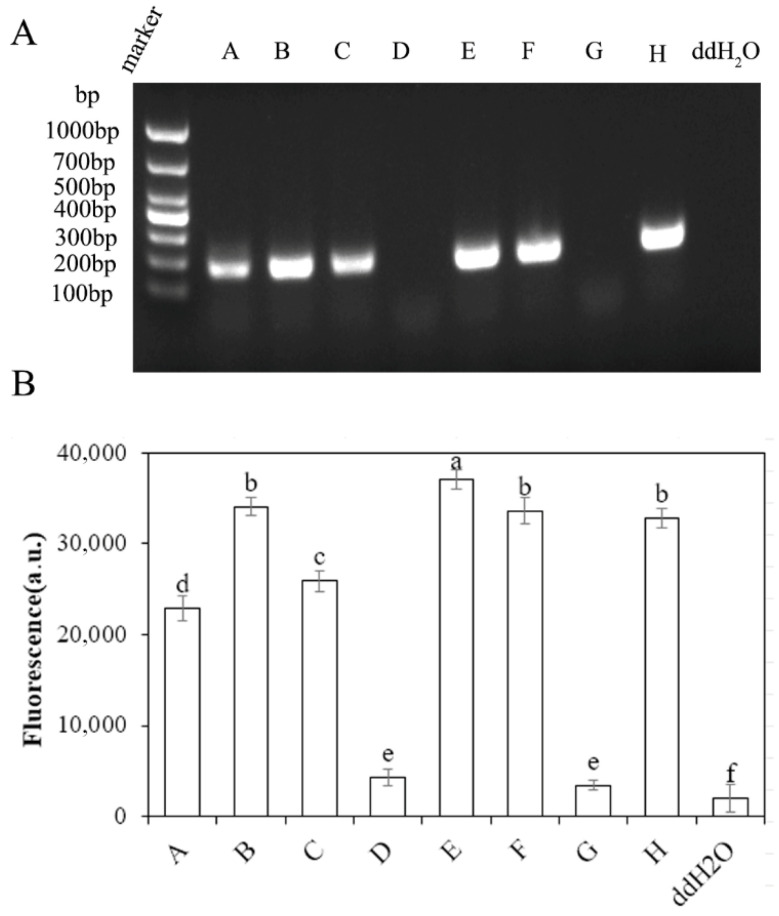
Selection and optimization of DNA extraction solutions. (**A**) PCR amplification of the target gene *Cglo6922* from *Cunninghamia lanceolata* using eight different extraction solutions (A–H). The composition of each extraction solution is provided in Table 1. (**B**) Quantitative analysis of the band intensities corresponding to the PCR products shown in (**A**). Band intensities were quantified using ImageJ software (version 1.54f, National Institutes of Health, Bethesda, MD, USA). Extraction solution E yielded the highest band intensity and was therefore selected as the optimal extraction solution for subsequent experiments. Different letters above the bars indicate statistically significant differences (*p* < 0.05).

To evaluate the efficiency of nucleic acid extraction using the EZ-D method and to select the optimal extraction solution, eight extraction solutions (Table 1) were tested for rapid nucleic acid extraction from *Cunninghamia lanceolata* using the EZ-D method. Conventional PCR amplification of the *Cunninghamia lanceolata* reference gene *Cglo6922* was performed using the primer pair *Cglo6922*F/R (Figure 2A). The results showed that the target fragment was amplified using extraction solutions A, B, C, E, F, and H, with extraction solution E yielding the highest band intensity. After comparing eight extraction buffers (Table 1), buffer E (TPS/CTAB extraction buffer, composed of 2% CTAB, 100 mM Tris pH 8.0, 1 M NaCl, and 10 mM EDTA) was identified as the optimal choice. Buffer E was uniformly used in all subsequent tests on actual samples.

In the comparative evaluation of the extraction methods (EZ-D strip protocol versus conventional CTAB method), the CTAB protocol was superior in terms of yield and purity, highlighting its effectiveness for high-precision DNA recovery (Table 2). However, the EZ-D strip method successfully extracted nucleic acids of *C. gloeosporioides* within only 1 min, whereas the CTAB protocol used in this study includes approximately 11–12 stages and can be completed in approximately 210 min. The cost per sample of the EZ-D strip method was also lower than that of the CTAB method. We have added an estimate of the total cost of the entire detection workflow (including EZ-D extraction, RPA amplification, and CRISPR/Cas12a detection). The results show that the total cost per sample using our method is approximately 1.2 RMB (about 0.17 USD), which is significantly lower than the 3.5 RMB (about 0.50 USD) required for the conventional CTAB method combined with PCR.

### 2.2. Identification of a Specific Target Gene for C. gloeosporioides

**Figure 3:**
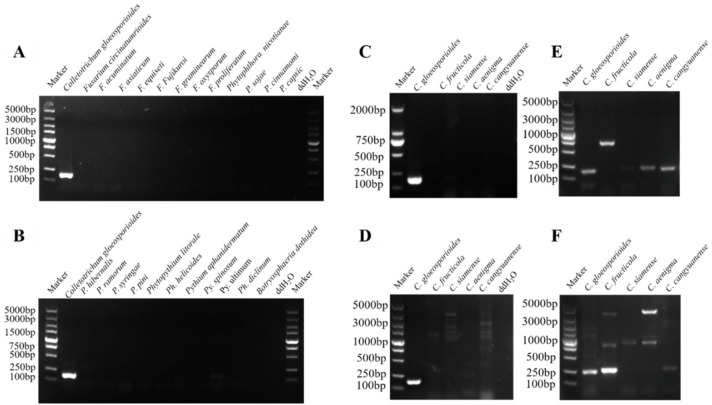
Specificity screening by conventional PCR assay. PCR products were separated by 1.5% agarose gel electrophoresis. Molecular weight markers: DL5000 and DL2000 (Takara Shuzo, Shiga, Japan). ddH_2_O was used as a negative control. (**A**–**C**) Primers targeting the *Cglo6922* gene. A specific amplicon of 197 bp was observed only in *C. gloeosporioides* samples, demonstrating specificity for *C. gloeosporioides* genomic DNA detection. (**D**–**F**) Primers targeting *Cglo2279* (**D**), *Cglo2375* (**E**), and *Cglo2856* (**F**), respectively. Non-specific amplification products were detected in other *Colletotrichum* isolates.

Conventional PCR screening of ten candidate target genes (Table S1) revealed that only the *Cglo6922*-specific primers (*Cglo6922*-PCR-F/R) generated a distinct 197 bp amplicon unique to *C. gloeosporioides*, without cross-reactivity against other *Colletotrichum* species or related genera (Figure 3). Conversely, the other nine primer sets exhibited non-specific amplification, such as multiple bands or smeared products. Consequently, *Cglo6922* was selected as the specific target for subsequent RPA-CRISPR/Cas12a detection.

### 2.3. Optimization of the RPA-CRISPR/Cas12a Detection System for C. gloeosporioides

**Figure 4:**
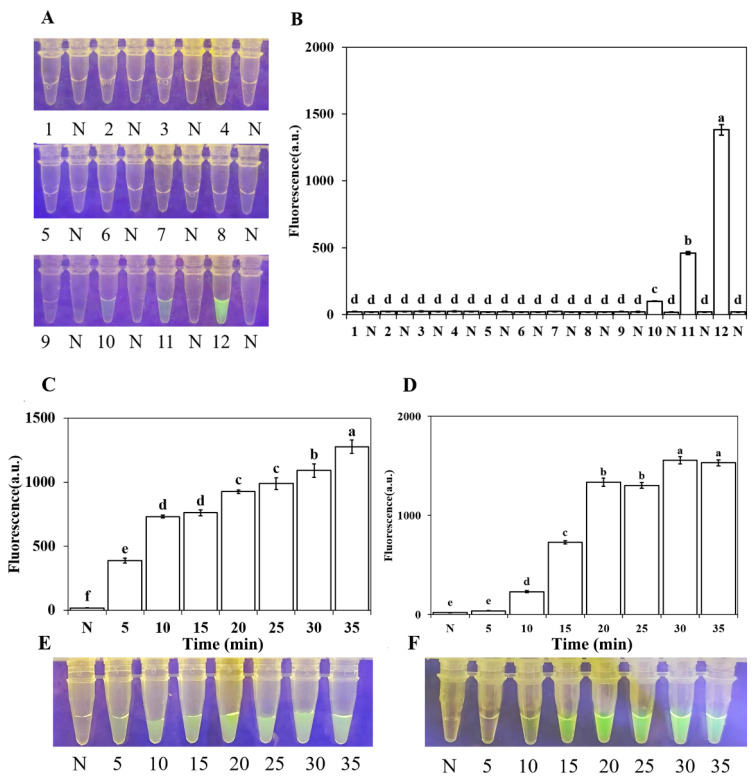
Optimization of crRNA and ssDNA reporter concentrations and reaction time in the RPA-CRISPR/Cas12a assay. (**A**,**B**) Optimization of crRNA and ssDNA concentrations. (**A**) Fluorescence intensity measured using a multimode microplate reader (λex: 485 nm, λem: 520 nm). Nos. 1–12 on the *x*-axis correspond to the 12 concentration combinations listed in Table 3. “N” indicates the negative control (no template). (**B**) Corresponding visual detection of green fluorescence under a blue LED fluorescence analyzer at 470 nm. (**C**,**E**) Optimization of RPA time. (**C**) Fluorescence intensity measured over different time points (5, 10, 15, 20, 25, 30, 35, and 40 min); the *x*-axis indicates time in minutes. (**E**) Corresponding visual detection of green fluorescence. (**D**,**F**) Optimization of Cas12a cleavage time. (**D**) Fluorescence intensity measured over different time points (5, 10, 15, 20, 25, 30, and 35 min). (**F**) Corresponding visual detection of green fluorescence. Fluorescence values were subjected to one-way analysis of variance (ANOVA). Different letters above the bars indicate statistically significant differences (*p* < 0.05).

To determine the optimal reaction conditions for the RPA-CRISPR/Cas12a system, different concentrations of crRNA and ssDNA reporter probe were tested. Fluorescence intensity analysis revealed that the optimal concentrations of crRNA and ssDNA reporter probe were 10 μM and 10 μM, respectively (Figure 4A,B). Optimization of the RPA reaction time (5, 10, 15, 20, 25, 30, 35, and 40 min) showed that green fluorescence could be observed under a blue light transilluminator as early as 5 min. However, the fluorescence intensity at 10 min reached a level statistically comparable to longer incubation times (15–40 min), with no significant signal increase beyond 10 min (Figure 4C). Therefore, 10 min was selected as the optimal RPA amplification time to balance rapid detection and sufficient sensitivity. The fluorescence intensity increased progressively with reaction time from 5 to 10 min but plateaued thereafter. Cas12a cleavage time tests (Figure 4C,D) indicated that 15 min was the optimal reaction duration. Taken together, the entire detection process could be completed within 25 min (10 min RPA amplification + 15 min Cas12a cleavage).

**Table 3:** CrRNA and ssDNA concentration combinations.

No.	CrRNA Concentration	ssDNA Concentration
1	40 nM	40 nM
2	80 nM	500 nM
3	300 nM	1.4 nM
4	0.5 μM	2 μM
5	0.6 μM	5 μM
6	1 μM	5 μM
7	2 μM	5 μM
8	5 μM	5 μM
9	1 μM	10 μM
10	2 μM	10 μM
11	5 μM	10 μM
12	10 μM	10 μM

### 2.4. Specificity of the RPA-CRISPR/Cas12a Detection Method for C. gloeosporioides

**Figure 5:**
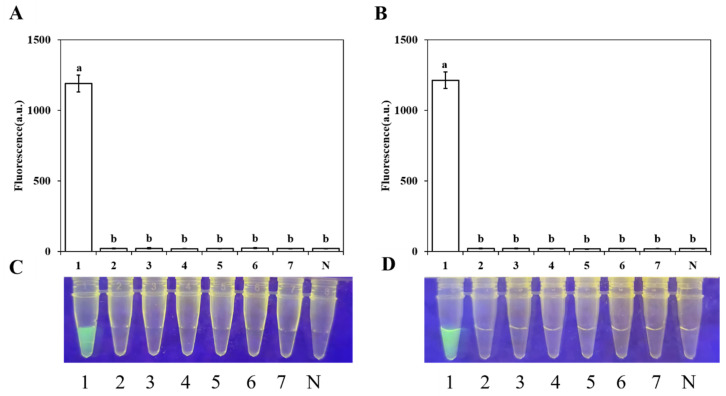
Specificity evaluation of the RPA-CRISPR/Cas12a detection assay. (**A**,**C**) Genomic DNA was evaluated from the following: 1: *C. gloeosporioides*; 2: *C. fructicola*; 3: *C. siamense*; 4: *C. aenigma*; 5: *C. cangyuanense*; 6: *C. metake*; 7: *C. karsti*; and N: no template. (**B**,**D**) Genomic DNA was evaluated from the following: 1: *C. gloeosporioides*; 2: *Fusarium solani*; 3: *F. acuminatum*; 4: *F. oxysporum*; 5: *F. proliferatum*; 6: *Phytophthora ramorum*; 7: *Botryosphaeria dothidea*; and N: no template. Fluorescence values were measured and analyzed by one-way ANOVA. Different letters indicate statistically significant differences (*p* < 0.05).

Specificity evaluation was performed using the strains listed in Table 4, including 15 *Colletotrichum* isolates derived from different hosts, 8 *Phytophthora* isolates, 6 *Phytopythium* isolates, 7 *Fusarium* isolates, and 1 *Botryosphaeria dothidea* isolate. As shown in Figure 5, a significant fluorescence signal was generated when *C. gloeosporioides* DNA was used as the template, whereas no fluorescence signal was observed for the other strains. Three independent replicate experiments yielded consistent results (*p* < 0.05), confirming the specificity of this system for *C. gloeosporioides*.

### 2.5. Sensitivity Determination of the RPA-CRISPR/Cas12a Detection Method

**Figure 6:**
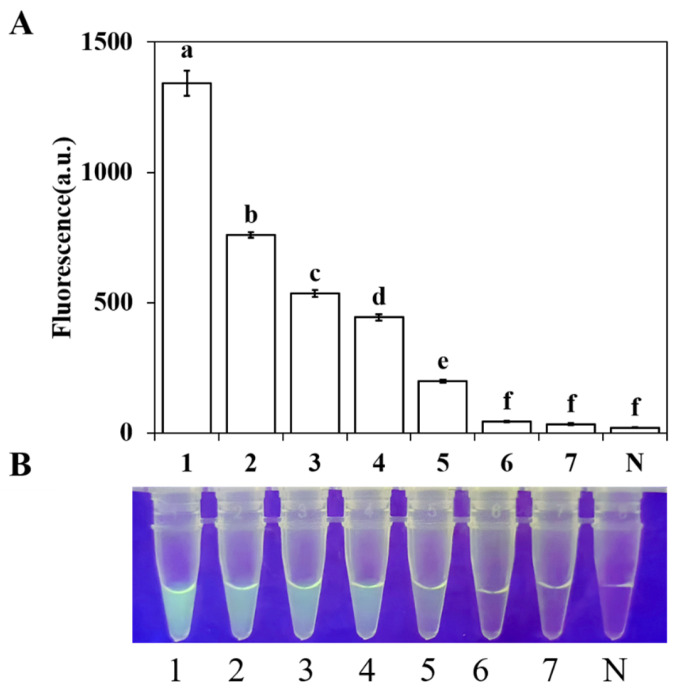
Sensitivity determination of the RPA-CRISPR/Cas12a detection system for *C. gloeosporioides*. The minimum detectable DNA concentration was 10 pg/μL. (**A**) Quantitative fluorescence measurement using a multimode microplate reader (λex: 485 nm, λem: 520 nm). (**B**) Visual detection of green fluorescence under a blue LED fluorescence analyzer at 470 nm. Lanes 1–8N: represent ten-fold serial dilutions of *C. gloeosporioides* gDNA: 1: 10 ng/μL; 2: 1 ng/μL; 3: 100 pg/μL; 4: 10 pg/μL; 5: 1 pg/μL; 6: 100 fg/μL; 7: 10 fg/μL; and N: ddH_2_O. Fluorescence values were subjected to one-way analysis of variance (ANOVA). Different letters above the bars indicate statistically significant differences (*p* < 0.05). Error bars represent standard deviations from three independent replicates.

The limit of detection (LOD) was evaluated by testing ten-fold serially diluted *C. gloeosporioides* genomic DNA (gDNA) ranging from 10 ng/μL to 10 fg/μL. The results showed that visible green fluorescence was generated when the gDNA concentration was ≥10 pg/μL, whereas no obvious fluorescence signal was observed for samples with concentrations ≤ 1 pg/μL or for the negative control (Figure 6). All results were consistent across three independent replicates. These findings demonstrate that the LOD of this system is 10 pg/μL. For each gDNA template concentration, the RPA-CRISPR/Cas12a assay was performed in triplicate under the same conditions as described above.

### 2.6. Detection of C. gloeosporioides in Artificially Inoculated Cunninghamia lanceolata Using the EZ-D-RPA-CRISPR/Cas12a System

**Figure 7:**
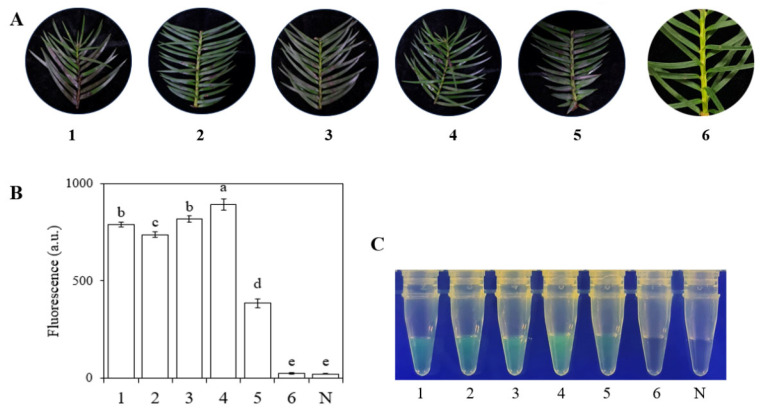
Detection of *C. gloeosporioides* in artificially inoculated *Cunninghamia lanceolata* branches using the RPA-CRISPR/Cas12a method. (**A**) Inoculated branch samples: 1–5, inoculated samples; 6, healthy branch; N, negative control (ddH_2_O). (**B**) Visual detection using a blue LED fluorescence analyzer (470 nm). Lanes: 1–5, samples at one week post-inoculation; 6, healthy branch; N, negative control. Different letters above the lanes indicate highly significant differences (*p* < 0.01) between fluorescent and non-fluorescent samples. (**C**) Quantitative fluorescence detection using a multimode microplate reader (λex = 485 nm, λem = 520 nm). Sample designations are the same as in (**B**). Error bars represent standard deviations from three independent replicates. Different letters indicate statistically significant differences (*p* < 0.05).

After inoculation and incubation, wilting symptoms appeared on the stems of *Cunninghamia lanceolata* leaves (Figure 7A). DNA extracted from the diseased leaves using the EZ-D method was subjected to RPA-CRISPR/Cas12a detection. As shown in Figure 7B,C, clear green fluorescence was observed in samples from infected *Cunninghamia lanceolata*, whereas no fluorescence signal was detected in the control samples or the no-template control (N). These results indicate that nucleic acids extracted via the EZ-D method are suitable for the detection of *C. gloeosporioides* in artificially inoculated *Cunninghamia lanceolata* using the RPA-CRISPR/Cas12a system. Consistent results were obtained across three independent replicate experiments.

## 3. Discussion

In this study, we established a method capable of detecting *C. gloeosporioides* within 30 min, from nucleic acid extraction to detection. Regarding nucleic acid extraction, the EZ-D method exhibited significant advantages over the conventional CTAB method. In terms of the time required to obtain amplifiable DNA from a single sample, the EZ-D method involved only two steps and took 30–60 s, whereas the CTAB method required 3 h. In addition to saving time and labor and improving efficiency, the cost of the EZ-D method was only CNY 0.2 (approximately $0.03 USD) per sample [2]. For large-scale sample screening in practical production, the cost would be approximately CNY 200 per 1000 samples. Other nucleic acid extraction methods, including silica membrane-based systems, polymer resin adsorption technologies, and magnetic bead-based protocols, also require longer processing times [49]. Furthermore, the EZ-D method requires less specialized expertise, fewer reagents, and simpler equipment [2]. Therefore, this method is accessible to a wider range of users and is suitable for broader applications, including field settings [50]. It also eliminates the need for toxic substances such as isoamyl alcohol, chloroform, and β-mercaptoethanol, which are required for the CTAB method [51,52,53]. Although the CTAB method yields higher quality DNA, and the EZ-D method produces less DNA with lower purity, the primary advantage of the EZ-D method lies not in absolute yield but in speed (30–60 s vs. >3 h), simplicity (no liquid nitrogen, no toxic chemicals), and field deployability. For applications requiring rapid on-site detection, the EZ-D method provides sufficient amplifiable DNA for RPA-CRISPR/Cas12a detection, as demonstrated in Figure 7 [2,54,55]. Owing to its numerous advantages, the EZ-D method merits widespread promotion and adoption.

The dual-recognition technology based on RPA and CRISPR-Cas12a enabled rapid and efficient detection, which could be visualized using a UV lamp or fluorescence dye [56]. During the experiments, we observed that the results were influenced by several factors, among which the concentrations of crRNA and ssDNA were critical for success. Specifically, insufficient concentrations of crRNA and ssDNA made the results difficult to interpret, whereas excessive concentrations increased costs [57]. To determine the most effective concentration combination, we tested 12 different combinations and ultimately concluded that the combination of 10 μM crRNA and 10 μM ssDNA minimized reagent waste while ensuring result visualization. The selection of a 10-min RPA amplification time in this study was based on systematic optimization (Figure 4C). Although visible fluorescence was detectable after only 5 min, the signal intensity at 10 min was statistically equivalent to that observed at longer incubation times (15–40 min), indicating that the amplification reaction had reached a plateau phase by 10 min. Extending the reaction beyond 10 min did not yield a significant improvement in fluorescence signal but would prolong the total assay time. Thus, 10 min was chosen as the optimal RPA reaction duration to achieve a rapid (25–30 min total) yet sensitive detection.

Under the same conditions, 10 other *Colletotrichum* species, 8 *Phytophthora* species, 6 *Phytopythium* species, 7 *Fusarium* species, and 1 *Botryosphaeria dothidea* isolate were tested. The results showed that fluorescence under blue light (470 nm) was observed only for *C. gloeosporioides*. By testing diseased tissues of artificially inoculated *Cunninghamia lanceolata* plants, we obtained accurate results, further confirming that this technique can effectively detect *C. gloeosporioides* in field environments. This finding has important implications for the prevention and control of *C. gloeosporioides*, as it provides a rapid and accurate detection method that facilitates timely detection and containment of the pathogen’s spread.

To provide a grounded quantitative comparison, our EZ-D-RPA-CRISPR/Cas12a system offers distinct advantages over existing methods for *C. gloeosporioides* detection. Conventional PCR typically requires 3–4 h from DNA extraction to result with a limit of detection (LOD) of approximately 1 ng/μL, and relies on thermal cyclers and gel electrophoresis apparatus. Real-time qPCR provides higher sensitivity (LOD ≈ 0.5 pg/μL) but still demands 2–3 h, expensive instrumentation, and skilled operators. LAMP-based methods reduce detection time to 60–120 min with an LOD of 10–100 pg/μL using simple heat blocks; however, they are prone to aerosol contamination and require complex primer design. In contrast, our method achieves an LOD of 10 pg/μL, completes the entire process within 25–30 min, operates at a constant 39 °C without any specialized equipment beyond a portable blue LED transilluminator, and costs approximately only USD 0.20 per sample. These quantitative advantages establish our method as a superior field-deployable point-of-care testing (POCT) tool. Compared with traditional PCR-based disease detection methods [58,59], this study provides a more efficient approach that does not require expensive instrumentation or highly specialized technical skills. Although many detection methods have been explored previously, including the combination of loop-mediated isothermal amplification (LAMP) with CRISPR/Cas12a for the development of novel diagnostic technologies, the integration of RPA with CRISPR/Cas12a offers a more effective tool for pathogen detection compared with other isothermal methods [60,61,62]. To date, some researchers have used LAMP methods to detect *C. gloeosporioides*, but the total time from sampling to result was approximately 120 min. In contrast, the RPA-CRISPR/Cas12a method substantially reduced the detection time.

In this study, we successfully developed an RPA-CRISPR/Cas12a-based detection method for the specific and rapid identification of *C. gloeosporioides*. Notably, this detection method does not require high technical expertise from the operator, does not rely on expensive instruments, and operates at a reaction temperature of 39 °C, which is well within the tolerable range for manual operation. These characteristics enhance convenience and safety throughout the detection process. By adopting this efficient and specific detection technique, early monitoring and timely warning of *C. gloeosporioides* can be achieved. This approach not only facilitates timely disease control measures to minimize economic losses but also contributes to the development of more scientifically sound disease prevention and control strategies. We acknowledge that the samples tested in this study were artificially inoculated under controlled conditions, and naturally infected field samples were not evaluated. This represents a current limitation. Nevertheless, artificial inoculation provided a controlled proof-of-concept that the EZ-D-RPA-CRISPR/Cas12a system can successfully detect *C. gloeosporioides* in plant tissue. Future studies are already underway to validate this method using naturally infected field samples collected from multiple geographical regions and host plants.

## 4. Materials and Methods

### 4.1. Rapid DNA Extraction Using the EZ-D Method

Genomic DNA was extracted from *Cunninghamia lanceolata* leaf tissue using the EZ-D method (Figure 1A). Approximately 50–100 mg of leaf tissue is used in the EZ-D method. This amount is substantially less than the 1 g required for the CTAB method, representing a major advantage of the EZ-D approach and making it suitable for rapid detection of limited sample amounts. To ensure the efficiency and success of the EZ-D method, eight extraction solutions were tested (Table 1). Leaf tissue was homogenized in 200 μL of extraction solution (100 mM Tris-HCl pH 8.0, 1 M NaCl, 10 mM EDTA) with two grinding beads (YMZ-S, 12.5 mm, stainless steel, Karryda, Zhuhai, China) by shaking the microcentrifuge tube manually or using a vortex mixer for 10 s. Subsequently, the EZ-D paper strip was immersed in the extraction buffer for 10 s. A purification step was performed in a bleaching solution (100 μL; 10 mM Tris-HCl pH 8.0, 0.1% Tween 20 (Solarbio, Beijing, China)) within a microcentrifuge tube to reduce potential PCR-inhibitory contaminants. The EZ-D strip was then placed into the amplification mixture and allowed to stand for 5 s. Through these three simple steps, amplifiable DNA was transferred from the extract to the amplification mixture via the EZ-D strip.

### 4.2. CTAB Extraction

The CTAB method was modified as follows [63]. Approximately 1 g of fresh leaf tissue was cut into 1–2 cm fragments, ground into a fine powder in a mortar containing approximately 50 mL of liquid nitrogen, and then transferred to a 1.5 mL microcentrifuge tube. CTAB-based lysis buffer (2% *w*/*v*, preheated at 65 °C for 30 min) was added to the sample matrix, followed by vigorous agitation to ensure homogeneous emulsification. The mixture was incubated with continuous agitation at 65 °C for 60 min, with inversion every 10 min to optimize cell lysis, and then centrifuged at 12,000× *g* for 10 min at 4 °C. The supernatant was carefully transferred to an analytical grade phenol-chloroform-isoamyl alcohol tube (25:24:1) for protein precipitation. This centrifugation step was repeated until phase separation was complete. The purified aqueous phase was subsequently transferred to a new microcentrifuge tube and precipitated at −20 °C for 1 h, followed by centrifugation at 12,000× *g* for 10 min at 4 °C. The pellet was washed twice with 70% ethanol (*v*/*v*) with gentle agitation, and then air-dried under sterile conditions. The dried nucleic acid pellet was resuspended in RNase A-treated ultrapure water (20 μg/mL) or TE buffer (pH 8.0), incubated at 37 °C for enzymatic digestion, and stored at −80 °C until use. The entire CTAB procedure, from tissue grinding to DNA elution, requires approximately 210 min.

### 4.3. Strains and Culture Conditions

Detailed information on the 15 *Colletotrichum* strains derived from different hosts, eight Phytophthora strains from different hosts, six *Phytopythium* strains from different hosts, seven *Fusarium* strains from different hosts, and one *Botryosphaeria dothidea* strain used in this study is presented in Table 3. All strains were deposited in the Department of Plant Pathology, Nanjing Forestry University. Their identities were confirmed by both morphological examination (colony morphology, conidial shape and size, and appressorium characteristics) and molecular identification using the internal transcribed spacer (ITS) region and partial glyceraldehyde-3-phosphate dehydrogenase (GAPDH) gene sequences, following the established taxonomic framework for the *C. gloeosporioides* species complex [64]. All sequence data were compared against the NCBI GenBank database using BLASTn to confirm species-level assignments. Fungal strains were grown on potato dextrose agar (PDA) at 25 °C in the dark for 3–5 days [65]. Oomycete strains were cultured on V8 juice agar at 18–25 °C in the dark [66]. Genomic DNA (gDNA) was extracted using the DNA Secure Plant Kit (Tiangen Biotech (Beijing) Co., Ltd., Beijing, China). The extracted DNA was quantified using a NanoDrop 1000C spectrophotometer (Thermo Fisher Scientific, Waltham, MA, USA) and appropriately diluted. All DNA samples were stored at −20 °C until use.

### 4.4. Design of RPA Primers, crRNA, and ssDNA Reporter Probe

To select candidate target genes specific to *C. gloeosporioides* from *Cunninghamia lanceolata* for the RPA-CRISPR reaction, the NCBI BLAST database (https://blast.ncbi.nlm.nih.gov/Blast.cgi, using the NCBI nr database, accessed on 15 May 2026) was searched against the genome sequences of *C. gloeosporioides*. To identify target genes unique to *C. gloeosporioides*, all publicly available genome sequences of *Colletotrichum* species were initially retrieved. Subsequently, all 26,131 gene sequences of *C. gloeosporioides* were used as queries to search against the aforementioned genomes using an e-value threshold of 1 × 10^−5^. Genes with no hits were considered unique to *C. gloeosporioides*. Ten candidate target genes were randomly selected, and primers were designed for conventional PCR amplification to screen for specific target genes (Table S1).

The *Cglo6922* gene was selected as the target for gene-specific RPA primer design (Figure S1). Before designing the primers and crRNA, we performed rigorous alignment validation of the selected target gene *Cglo6922* and its primer sequences using the NCBI BLAST database. Using the *Cglo6922* gene sequence as a query, a BLASTn search was conducted against GenBank. The results showed that this gene shares highly homologous sequences only with *C. gloeosporioides*, with no significant matches to other *Colletotrichum* species or non-target fungi. The RPA primers and the crRNA targeting region were also validated by BLAST to ensure their sequence specificity. RPA primers were designed using Primer Premier 6.0 software (Premier Biosoft, San Francisco, CA, USA) following the manufacturer’s recommendations for RPA primer design (Table 5). The crRNA and ssDNA reporter probe were designed using the CHOPCHOP online tool [67]. The crRNA sequence did not overlap with the RPA primers and targeted a conserved region within the RPA amplicon (Table 4). The ssDNA reporter probe was labeled with a 6-FAM fluorophore at the 5′ end and a BHQ-1 quencher at the 3′ end. Both the crRNA and ssDNA reporter probe were synthesized by GenScript Biotech (Nanjing, China) and stored at −20 °C until use [66].

### 4.5. Conventional PCR Assay

Conventional PCR was performed in a 50 μL reaction mixture containing 25 μL of PrimeSTAR Max PreMix 2× (Takara Bio, Dalian, China), 21 μL of ddH_2_O, 2 μL of purified gDNA (100 ng·μL^−1^), and 1 μL each of forward and reverse primers (10 μM) (Table 4) [68]. The thermal cycling program was as follows: initial denaturation at 94 °C for 3 min; followed by 33 cycles of denaturation at 94 °C for 30 s, annealing at 60 °C for 30 s, and extension at 72 °C for 45 s; with a final extension at 72 °C for 10 min. The PCR products were analyzed by electrophoresis on a 1.5% agarose gel [66].

### 4.6. Establishment of the RPA-CRISPR/Cas12a Detection System

Step 1 (RPA): A 50 μL RPA reaction was prepared using the Test Strip Kit (Lesheng Biotechnology, Wuxi, China). Each reaction mixture contained 2 μL of forward and reverse primers (10 μM each), 25 μL of buffer, 2 μL of template DNA (100 ng/μL), and 16 μL of ddH_2_O. After centrifugation at 4000 rpm for 5 s, 3 μL of the initiator (provided with the kit) was added to the tube cap. The reaction unit was then sealed, inverted 8–10 times, and briefly centrifuged at 4000 rpm to ensure thorough mixing of the solution with the initiator. The reaction was incubated at 39 °C for 4 min, followed by a second inversion mixing and brief centrifugation, and then incubated for an additional 16 min. The amplification products were subsequently analyzed using the CRISPR/Cas12a system.

Step 2 (CRISPR/Cas12a detection): The ssDNA reporter probe, labeled with 6-FAM/BHQ-1, was added to the CRISPR/Cas12a reaction mixture. Upon specific recognition of the target protospacer sequence by the Cas12a/crRNA complex, the non-specific ssDNA trans-cleavage activity of Cas12a was activated, resulting in the release of a fluorescent signal. Gradient concentration tests were performed to determine the optimal concentrations of crRNA and ssDNA reporter probe (crRNA: 40 nM, 80 nM, 300 nM, 0.5 μM, 0.6 μM, 1 μM, 2 μM, 5 μM, 10 μM; ssDNA reporter probe: 1.4 nM, 40 nM, 500 nM, 1 μM, 2 μM, 5 μM, and 10 mM) (Table 3). Meanwhile, seven time gradients (5, 10, 15, 20, 25, 30, and 35 min) were tested to optimize the RPA and Cas12a cleavage times, respectively. The final 50 μL CRISPR reaction mixture consisted of 38 μL of ddH_2_O, 5 μL of reaction buffer (10× Buffer), 3 μL of crRNA, 1 μL of Cas12a enzyme, 1 μL of ssDNA reporter probe, and 2 μL of RPA product. The mixture was centrifuged at 4000 rpm for 5 s and then incubated at 39 °C.

The results of the RPA-CRISPR/Cas12a assay were evaluated using two different methods: fluorescence intensity was measured using a multimode microplate reader (λex 485 nm, λem 520 nm), or green fluorescence was visualized under a blue LED transilluminator at 470 nm wavelength. Negative controls exhibited no fluorescent signal. All RPA-CRISPR/Cas12a experiments were performed in triplicate. The standard deviation of the three replicates was calculated using the STDEVP function. Statistical analysis was conducted using GraphPad Prism 8 (GraphPad Software Inc., San Diego, CA, USA), and a *p*-value < 0.05 was considered statistically significant.

### 4.7. Specificity and Sensitivity of the RPA-CRISPR/Cas12a Detection Method

To evaluate specificity, the strains listed in Table 3 (including 15 *C. gloeosporioides* isolates, 8 *Phytophthora* isolates, 6 *Phytopythium* isolates, 7 *Fusarium* isolates, and 1 *Botryosphaeria dothidea* isolate) were used. To determine sensitivity, purified gDNA of *C. gloeosporioides* at concentrations ranging from 100 ng, 10 ng, 1 ng, 100 pg, 10 pg, 1 pg, to 100 fg was used as the DNA template in the RPA-CRISPR/Cas12a reaction system, with 100 ng of purified gDNA as the positive control and ddH_2_O as the no-template control (N). All RPA-CRISPR/Cas12a reactions were performed in triplicate. The results from the three replicates of the CRISPR/Cas12a assay were analyzed using the STDEVP function to calculate the standard deviation. Statistical analysis was performed using GraphPad Prism 8 software (GraphPad Software Inc., San Diego, CA, USA), and a *p*-value < 0.05 was considered statistically significant.

### 4.8. Application of the RPA-CRISPR/Cas12a Method for Detection of C. gloeosporioides in Artificially Inoculated Cunninghamia lanceolata

*C. gloeosporioides* was cultured on PDA medium at 25 °C in the dark for 3 days. To prepare the conidial suspension, 5 mL of sterile distilled water containing 0.05% Tween-20 was added to the culture plate. The colony surface was gently scraped with a sterile spatula to dislodge conidia. The resulting suspension was filtered through three layers of sterile gauze or a 40-μm cell strainer to remove mycelial debris. The filtrate was collected and centrifuged at 5000× *g* for 5 min, and the supernatant was discarded. The conidial pellet was then washed twice with sterile distilled water by repeated centrifugation and resuspension. After the final wash, the conidia were resuspended in sterile distilled water, and the concentration was adjusted to 1 × 10^6^ conidia·mL^−1^ using a hemocytometer.

The conidial suspension was then evenly sprayed onto the surface of treated detached *Cunninghamia lanceolata* branches, which were subsequently maintained under humid conditions. Obvious lesions were observed on the branches at 3 days post-inoculation. At 4 days post-inoculation, DNA was extracted from branch tissues of both inoculated and healthy (non-inoculated) plants using the method (EZ-D) described in Section 2.1. The RPA-CRISPR/Cas12a detection was then performed following the procedure illustrated in Figure 1. Sterile water was used as a negative control. A positive result was indicated by the presence of green fluorescence, whereas no fluorescence was observed for negative results. Fluorescence was visualized under a blue LED transilluminator at 470 nm or measured using a multimode microplate reader. Each detection method was performed in triplicate.

## 5. Conclusions

In this study, we have developed a rapid, specific, and sensitive detection method for *C. gloeosporioides* based on RPA-CRISPR/Cas12a combined with the EZ-D rapid nucleic acid extraction technique. The entire detection process can be completed within 25 min without the need for expensive instrumentation or highly specialized technical expertise. Integrative evaluation shows that the method achieves a limit of detection of 10 pg/μL genomic DNA and exhibits no cross-reactivity against 32 non-target isolates, demonstrating robust species-level specificity, while the total cost per sample is approximately 1.2 RMB (about 0.17 USD), significantly lower than that of conventional methods. In positioning within existing technologies, compared to traditional PCR, qPCR, and LAMP-based methods, our approach offers a better balance of speed, simplicity, and field deployability, making it particularly suitable for point-of-care testing (POCT) in resource-limited settings. Regarding remaining challenges for practical implementation, future work should focus on: (1) more convenient result readout methods (e.g., lateral flow strips); (2) long-term preservation strategies for field samples; (3) development of multiplex detection capability for simultaneous identification of multiple pathogens; and (4) validation with naturally infected field samples from diverse geographical regions and host plants.

## Figures and Tables

**Table 1:** Composition of eight tested extraction mixtures.

Extract	Extraction Mixture Name	Composition
A	TPS extract	100 mM Tris pH 8.0, 1 M NaCl, 10 mM EDTA
B	SDS extract	20 mM Tris pH 8.0, 25 mM NaCl, 2.5 mM EDTA, 0.05% SDS
C	CTAB extract	2% CTAB, 50 mM Tris pH 8.0, 0.7 M NaCl, 10 mM EDTA
D	TPS/SDS extract	0.005% SDS, 100 mM Tris pH 8.0, 1 M NaCl, 10 mM EDTA
E	TPS/CTAB extract	2% CTAB, 100 mM Tris pH 8.0, 1 M NaCl, 10 mM EDTA
F	0.1 M NaOH solution	0.1 M NaOH
G	Optimized NaOH extract	0.1 M NaOH, 0.05% SDS, 2% PVP
H	Tween-20 extract	50 mM Tris pH 8.0, 150 mM NaCl, 2% PVP, 1% Tween 20

Note: All percentages are *w*/*v*.

**Table 2:** DNA extracted from *Cunninghamia lanceolata* leaves using CTAB and EZ-D methods.

Method	DNA Concentration (ng/μL)	A_260_/A_280_	A_260_/A_230_	Total Time Cost	Personal Skill	Organic Reagent	Electrical Device	Cost Per Sample (CNY)
CTAB	217.59 ± 3.73 ^a^	1.73 ± 0.07 ^a^	1.77 ± 0.01 ^a^	1.5–3.5 h	High	Yes	Yes	0.9
EZ-D (Extract A)	28.1 ± 2.34 ^e^	1.67 ± 0.08 ^b^	0.25 ± 0.02 ^c^	30–60 s	Low	No	No	0.2
EZ-D (Extract B)	35.93 ± 1.78 ^c^	1.45 ± 0.09 ^c^	0.34 ± 0.03 ^b^	30–60 s				0.2
EZ-D (Extract C)	30.1 ± 2.98 ^d^	1.33 ± 0.03 ^d^	0.27 ± 0.04 ^c^	30–60 s				0.2
EZ-D (Extract D)	17.74 ± 4.23 ^f^	1.37 ± 0.04 ^d^	0.15 ± 0.05 ^d^	30–60 s				0.2
EZ-D (Extract E)	37.21 ± 3.24 ^b^	1.28 ± 0.02 ^d^	0.31 ± 0.06 ^b^	30–60 s				0.2
EZ-D (Extract F)	36.96 ± 2.19 ^c^	1.04 ± 0.03 ^e^	0.25 ± 0.07 ^c^	30–60 s				0.2
EZ-D (Extract G)	16.89 ± 3.63 ^g^	1.18 ± 0.04 ^e^	0.35 ± 0.08 ^b^	30–60 s				0.2
EZ-D (Extract H)	34.52 ± 3.87 ^c^	1.63 ± 0.05 ^b^	0.30 ± 0.05 ^b^	30–60 s				0.2

Note: Different letters after the means in a column indicate a significant difference in a one-way ANOVA followed by Tukey’s HSD test (*p* <  0.05).

**Table 4:** Information and CRISPR-Cas12a detection results of *C. gloeosporioides* and other oomycete/fungal isolates used in this study.

Number	Species	Strain	Host	Source	RPA-CRISPR/Cas12a Detection Result
1	*C. gloeosporioides*	SMCG1	*C. Lanceolata*	JS	+
2	*C. gloeosporioides*	GH7	*Osmanthus fragrans*	AH	+
3	*C. gloeosporioides*	QFRH01	*Cunninghamia lanceolata*	JS	+
4	*C. metake*	YB1	*Chimonobambusa quadrangularis*	JS	−
5	*C. fructicola*	YB2	*C. quadrangularis*	JS	−
6	*C. karsti*	YB3	*C. quadrangularis*	JS	−
7	*C. plurivorum*	YB4	*C. quadrangularis*	JS	−
8	*C. cliviae*	YB5	*C. quadrangularis*	JS	−
9	*C. boninense*	YB6	*C. quadrangularis*	JS	−
10	*C.graminicola*	YB7	*C. quadrangularis*	JS	−
11	*C. fructicola*	MQ11-3	*Cunninghamia lanceolata*	FJ	−
12	*C. siamense*	YK102	*Cunninghamia lanceolata*	FJ	−
13	*C. siamense*	YK38	*Cunninghamia lanceolata*	FJ	−
14	*C. aenigma*	WH2-9	*Fragaria × ananassa Duch.*	FJ	−
15	*C. cangyuanense*	SR9	*Cunninghamia Lanceolata*	FJ	−
16	*Fusarium circinatum*	A045-1	*Pinus* sp.	JS	−
17	*F. acuminatum*	Fac1	*Rhizophora apiculate*	SC	−
18	*F. asiaticum*	Fas1	*Triticum aestivum*	JS	−
19	*F. equiseti*	Feq1	*Glycine max*	JS	−
20	*F. Fujikuroi*	Ffu1	*Oryza sativa*	JS	−
21	*F. graminearum*	Fgr1	*Triticum aestivum*	JS	−
22	*F. oxysporum*	Fox2	*Pinus* sp.	JS	−
24	*Phytophthora nicotianae*	Pn1	*Nicotiana tabacum*	FJ	−
25	*P. sojae*	P6497	*Glycine max*	USA	−
26	*P. cinnamomi*	23B2	*Persea americana*	Puerto Rico	−
27	*P. capsici*	Pcap1	*Capsicum annuum*	JS	−
28	*P. hibernalis*	947	*Citrus sinensis*	SH	−
29	*P. ramorum*	EU1 2275	*Quercus palustris*	United Kingdom	−
30	*P. syringae*	9099	*Citrus reticulata Blanco*	SH	−
31	*P. pini*	Ppini	*Rhododendron pulchrum*	JS	−
32	*Phytopythium litorale*	PC-dj1	*Rhododendron simsii*	JS	−
33	*Ph. helicoides*	PH-C	*Rhododendron simsii*	JS	−
34	*Pythium aphanidermatum*	NT-ap1	*Nicotiana tabacum*	JS	−
35	*Py. spinosum*	OS-sp1	*Oryza sativa*	JS	−
36	*Py. ultimum*	GM-ul1	*Glycine max*	JS	−
37	*Ph. diclinum*	RS-di1	*Rhododendron simsii*	JS	−
38	*Botryosphaeria dothidea*	Bci1	*Koelreuteria paniculata*	JS	−

Note: Abbreviations for Chinese provinces, municipalities, and regions: AH = Anhui; FJ = Fujian; JS = Jiangsu; SC = Sichuan; SH = Shanghai. Positive and negative results for RPA-CRISPR/Cas12a are denoted by “+” and “−”, respectively.

**Table 5:** Oligonucleotides and target gene sequence used in this study.

Item	Sequence (5′→3′)
Target gene (*Cglo6922*)	ATGGGCAAGATCTACACCGACTGTACGAACGGCCTGGTATGGCTGGGAGACTTGGAGTCTAATCTGAACGATTCCGTGTTTGTCGAAGCTCACCGCGAATGCTGGTCCTTCGCGGGTGACTTCAGTGACTACGAAAGACTGTGGGCTAGGCTTGTCAAAGTGTTAGGCATCACACGTCGCGAGTCAGCCAGTGTCAAAAGATGCACGTCCAATGACGCTTTCAAGGGCGAAGAACGGATTCTCGAAGTTGAGTGGGCCAATTACGCAAATCGCCTGTCTTTAGGAGAGTCTGCCTTTCACTTAGCATGGTTTCTTCGGCAACTTCCTGAACGATACCCAAGCCGGCGCACAACAGACCCCTACATGAACTTACCGCATCCTCTGTTCGGCTCATCTTGGGTGGAGGGAAACTGGTCACCGTATTGGGAATCTATCTTGAACGTACTCGCCATCGTTGTTCGAGATCCATGGTGGCGCAGGCTCTGGGTAGTCCAGGAGTTGGCCCTTCCCCCCGATGTTCACTTCCTATTCGGCCCTGTCGCGATTTCCCTCGATATATTCACGGATGCGCTGTACTTCATGCAGAACTGCAATATTTTCAACCGCTACTCCAGCCGCGAAGGCGATGAGATTCTGGACCTAGCTTCCCGGCATTGGAAATAA
Conventional PCR primers	F1: TACTCGCCATCGTTGTTC
	R1: CCAGAATCTCATCGCCTTC
RPA primers	F1: CGTACTCGCCATCGTTGTTCGAGATCCATGGTGG
	R1: CCAGAATCTCATCGCCTTCGCGGCTGGAGTAGCG
crRNA	UAAUUUCUACUAAGUGUAGAUCCUCGAUAUAUUCACGGAUG
ssDNA reporter	6-FAM-TTATT-BHQ-1

Note: FAM, 6-carboxyfluorescein; BHQ-1, Black Hole Quencher-1.

## Data Availability

The raw data supporting the conclusions of this article will be made available by the authors on request.

## Supplementary Materials

The following supporting information can be downloaded at: https://www.mdpi.com/article/10.3390/plants15101565/s1. Figure S1. Target sequence design for CRISPR-Cas mediated gene editing in *C. gloeosporioides*. Table S1. Candidate target genes and RPA primer sequences for CRISPR-Cas detection in *C. gloeosporioides*.
