# Supplementary figures and images for: Rapid On-Site Detection of Colletotrichum gloeosporioides Using EASY DNA Extraction (EZ-D) Method Combined with RPA-CRISPR/Cas12a
Source: Plants (Basel). 2026 May 20;15(10):1565. doi: 10.3390/plants15101565 (PMC13210964; doi:10.3390/plants15101565)

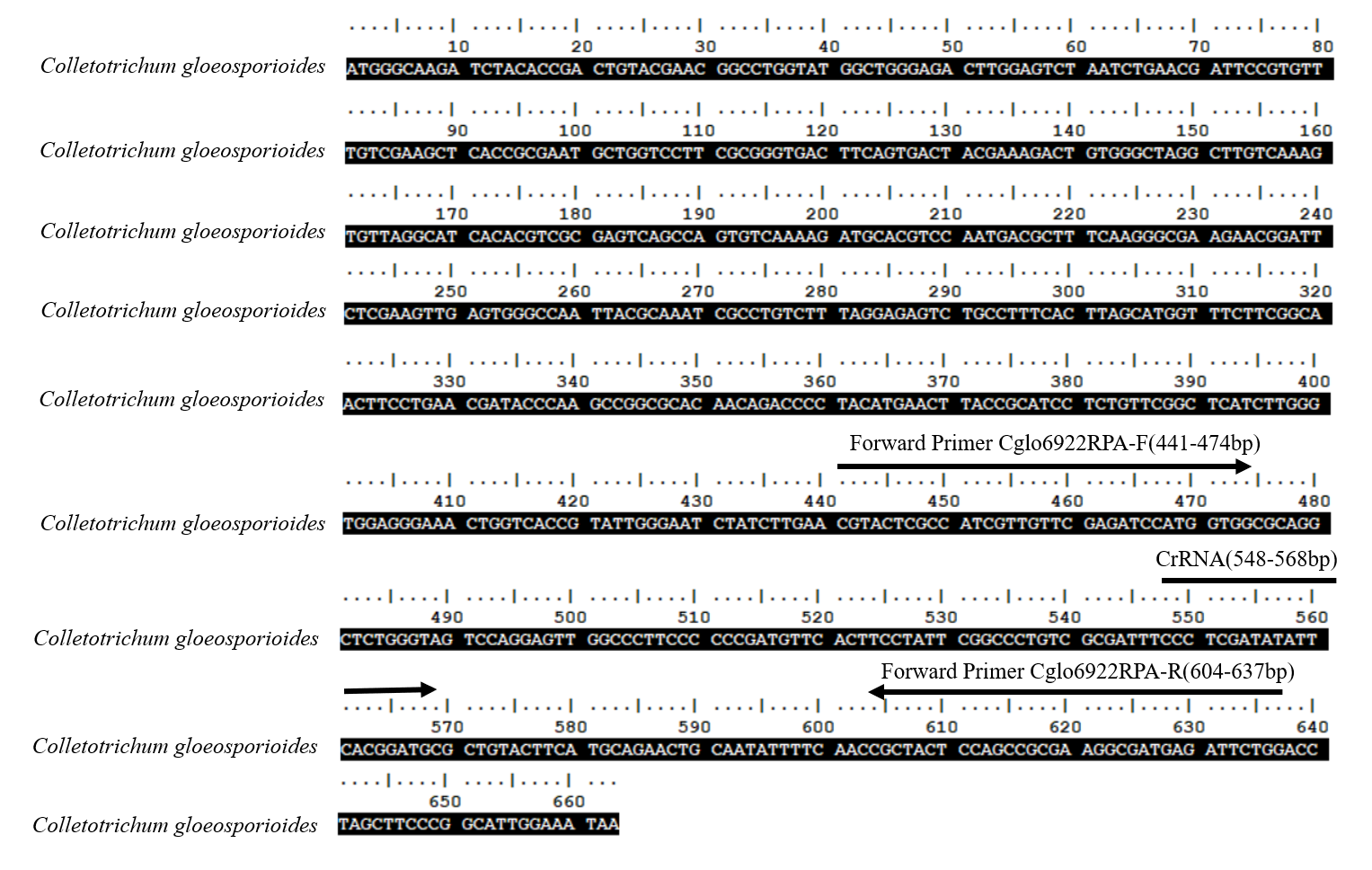

Supplement: Supplementary file 1 [file plants-15-01565-s001.zip › Figure S1.tif]
